# Genome-wide variation in and between two closely related underutilised horsegram species (*Macrotyloma axillare* and *M. uniflorum*, Fabaceae)

**DOI:** 10.1093/aobpla/plag003

**Published:** 2026-01-16

**Authors:** Niall P Taylor, Mark A Chapman

**Affiliations:** Biological Sciences, University of Southampton, Life Sciences Building 85, Highfield Campus, Southampton, Hants, SO17 1BJ, United Kingdom; Biological Sciences, University of Southampton, Life Sciences Building 85, Highfield Campus, Southampton, Hants, SO17 1BJ, United Kingdom

**Keywords:** genetic variation, horsegram, *Macrotyloma*, underutilised crop

## Abstract

The assessment of the degree and partitioning of genetic variation in crop populations and species is crucial to understand their adaptive evolution and provides vital knowledge to assist in the development of crops to combat food insecurity. Underutilised crops are understudied but are often drought-/heat-tolerant or nutritionally diverse; hence, as food security becomes more pressing, their investigations are increasing. Here, we focus on horsegram (*Macrotyloma uniflorum* (Lam.) Verdc.) and perennial horsegram (*M. axillare* (Meyer) Verdcourt), two closely related drought- and heat-tolerant underutilised legumes. Forty-two accessions were studied through phylogenetic and population genetic analysis and by measuring their seed and plant morphologies to assess genetic and morphological variation within and between the species. The species were distinct at the genetic level, with genetic diversity about 2.5 times greater in *M. axillare* than in *M. uniflorum*. Previously unsampled horsegram accessions from Africa were distinct from South Asia and therefore could contain novel genetic variation. Genetic variation suggested four clusters within perennial horsegram, which were largely structured by geography. Seed length is significantly greater in horsegram, and the two species differ in their dominant seed and stem colours, which could assist in-field identification. This work provides new insight into these species specifically and underutilised legumes more generally. Future investigations focused on identifying adaptive genetic variation are warranted to further reveal the potential of these crops in being optimized for promotion and commercialization, especially in countries which need more sustainable and reliable agricultural varieties to mitigate climate change.

## Teaser

Understanding the extent and partitioning of morphological and genetic variation in crops and their close wild relatives helps to identify novel variation that could be used in crop improvement. In our work we investigated two closely related underutilised legume crops called horsegram and perennial horsegram. We show that the species are morphologically similar, but a suite of characters can be used to differentiate them. Genetically the species are distinct; cultivated samples are closely related to each other, but wild samples of each are genetically variable. Wild samples from Africa in particular are the most variable, and novel adaptive variation may be present in these.

## Introduction

Currently, agriculture is facing challenges from global warming and an increasing frequency of erratic and extreme weather events (IPCC 2021), with implications for population health and wellbeing (Ebi et al. 2021). This warming has consequently exceeded the threshold for exposure to climate-tipping point triggers, defined as irreversible and abrupt changes to the climate system (Armstrong McKay et al. 2022) resulting in periods of extended drought and extreme heat of which many vulnerable countries and areas would be agriculturally decimated. Many commercial crops have been highlighted to have high probability of yield losses if exposed to extreme heat and drought events (Leng and Hall 2019). This is being realized in some locations and for some crops (Tesfaye et al. 2015, McCarthy et al. 2021). As a result, value lies in the research of drought- and heat-resistant crops to reduce the risk of food insecurity in at-risk areas.

Underutilised crops are domesticated plant species which have been neglected from a research standpoint and have not been the focus of commercialization yet offer future potential in a changing climate facing food insecurity (Mayes et al. 2012, Shorinola et al. 2024). These crops are currently relied upon by smaller scale operations such as local farming (Ochatt and Jain 2007). Their description as underutilised unites these crops as demonstrating traits which are agriculturally favourable such as being rich in protein, macro- or micronutrients, or pharmaceuticals as well as providing drought- or other stress resilient traits (Abberton et al. 2022). Underutilised legume crops, in particular, have been voiced as pivotal for future food security because they are not only rich in protein and environmentally adaptable, but they can fix atmospheric Nitrogen, therefore reducing the need for environmentally harmful nitrogen-based fertilizer (Zahran 1999). Underutilised crops are commonly neglected from agricultural practises due to negative cultural perception (e.g. as a ‘famine food’), unrecognized nutritional potential and/or irregular consumption/growth requirements (Williams and Haq 2000).

Understanding the genetic structure of a species allows for identifying untapped germplasm and novel alleles, potentially involved in local adaptation or other adaptive traits (Voss-Fels and Snowdon 2016). For underutilised crops, often limited or no such studies have been carried out, although this situation is changing with molecular marker studies (e.g. Yang et al. 2018, Uba et al. 2021) and reference genomes and population genomic studies recently in several underutilised crops (Chapman et al. 2022).

Here we focus on two closely related horsegram species in genus *Macrotyloma* (*M. uniflorum* [horsegram] and *M. axillare* [perennial horsegram]; Fabaceae), a genus which also contains a third species grown for food (*M. geocarpum* (Harms) Maréchal & Baudet). *Macrotyloma* species are drought- and heat-tolerant and have been recognized as having potential in a changing climate (Aditya et al. 2019). As members of subtribe Phaseolinae they are related to genera which contain many edible legumes of varying global importance, such as the widespread common bean (*Phaseolus vulgaris* L.), more localized lima bean (*P. lunatus* L.), Tepary bean (*P. acutifolius* A. Gray), cowpea (*Vigna unguiculata* (L.) Walp.), mung bean (*V. radiata* (L.) Wilczek) and black gram (*V. mungo* (L.) Hepper), and the relatively minor species adzuki bean (*V. angularis* (Willd.) Ohwi & H. Ohashi), moth bean (*V. aconitifolia* (Jacq.) Maréchal), rice bean (*V. umbellata* (Thunb.) Ohwi & H. Ohashi), zombi pea (*V. vexillata* (L.) A. Rich.), Bambara groundnut (*V. subterranea* (L.) Verdc.), and lablab (*Lablab purpureus* (L.) Sweet).

Horsegram is an important, albeit minor legume in cuisine in India and other parts of South Asia (Chahota et al. 2013, Fuller and Murphy 2018), whereas perennial horsegram is cultivated on a minor scale and solely as forage (Blumenthal and Staples 1993). Horsegram is nutritious, with 57.2% carbohydrates (of which 16.3% is dietary fibre), 16%–30% protein and 0.5% fat, therefore assists with proper gut functioning and potentially can help reduce chronic diseases such as heart disease in humans (Yadav et al. 2004, Bhartiya et al. 2015). Horsegram is found in Africa and Southeast Asia with the latter representing the cultivated type. Perennial horsegram is so abundant in nitrogen and protein that it is one of a small number of forage legumes considered well above adequate for the requirements of growing beef cattle (Valarini and Possenti 2006). Despite these attributes, both species remain underutilised. While improved horsegram varieties have been released (Chahota et al. 2013), it is often perceived as a ‘famine food’ and perennial horsegram is often targeted with herbicides because it is thought of more as a weed than a forage in some places (Weiss et al. 2004).

Further investigation of the species, especially their genetic variation, could therefore be a catalyst in changing the perception of both legumes and enhancing the farming of them as crops. Investigations of horsegram are much more advanced than those into perennial horsegram. Two genome sequences have been generated for horsegram (Mahesh et al. 2021, Shirasawa et al. 2021), both covering about 85%–90% of the genome; however, only one has been assembled into pseudochromosomes (Shirasawa et al. 2021), which was done using a mapping population. The only genome sequence for perennial horsegram was generated using short reads and therefore is fragmented and was only used to identify molecular markers (Fisher et al. 2022). Nevertheless about 75% of the genome was estimated to be covered and 85% of the gene space.

Genetic variation in horsegram suggests a low level of genetic diversity with relatively small numbers of polymorphic single-nucleotide polymorphisms (SNPs) uncovered in diversity panels (e.g. <300 SNPs in the study by Shirasawa et al. (2021) using an approach that typically resolves thousands of markers). These panels, however, only considered accessions from India, whereas wild populations of the species are also found in Africa.

Phylogenetic analysis based on three chloroplast (cp) DNA barcodes places the two horsegram species in the same clade but not reciprocally monophyletic (Fisher et al. 2022) raising the question as to whether these species are distinct or simply represent annual and perennial forms of the same taxon; this is sometimes suggested for perennial *Oryza rufipogon* and annual *O. nivara*, the closest wild relatives of cultivated rice (Morishima et al. 1961, Zheng and Ge 2010).

The aims of this work were to (i) determine whether the two species are reciprocally monophyletic, (ii) analyse population structure and quantify genetic diversity within each species, and (iii) compare the morphologies of seed and adult plant characters to help identify characters to differentiate the species. We employed a reduced-representation sequencing approach such that the genome-wide genetic diversity could be assessed using multiple genetic markers (SNPs), and we extended previous sampling where possible to include accessions from outside South Asia.

## Materials and methods

### Seed samples

Seed were obtained from a range of seed banks for both species and two outgroup species, *M. africanum* and *M. daltonii* (identified from Fisher et al. 2022), with the goal of sampling across the species’ ranges of both horsegram and perennial horsegram (Supplementary Table S1). In total, 17 samples of *M. uniflorum*, 25 of *M. axillare*, two of *M. africanum* and one of *M. daltonii* were utilized.

### Morphological analysis

Five seed per accession were chipped using a razor blade and placed in pots containing 1:1 Levington’s M2 + S:vermiculite. Pots were bottom watered daily in a greenhouse with a minimum temperature of 22°C (although often hotter during the day) and a 16-hour daylength. After germination three plants per accession were retained and separated into different pots. Morphological analysis was carried out on the seed directly obtained from the seed banks and the three plants. The seed length was quantified (to the nearest 0.01 mm) on five seeds per accession (chosen randomly) and the dominant seed colour noted (some accessions were heterogeneous for seed coat colour; however, there was usually one dominant colour). Leaf and leaflet width were quantified (Supplementary Figure S1) for the second, fifth, and eighth leaves on each stem of the plant and the mean per plant calculated. Stem colour was noted for all plants; where colour varied along the stem within individuals, again the dominant colour was recorded.

### Genetic analysis

The SNPs were obtained using a reduced representation sequencing approach, SLAF-seq (specific locus amplified fragment sequencing (Sun et al. 2013), in which restriction enzymes digest the genome into fragments which are PCR amplified and sequenced (Zhou and Pan 2023).

DNA was extracted from a single randomly selected individual of each accession using a CTAB-based approach (Doyle and Doyle 1990). Samples were quantified and examined for quality using a Nanodrop and gel electrophoresis. DNA was sent to BMKGENE (Alderley Park, UK) for SLAF-seq. After a pre-experiment, the enzymes *Rsa*I + *Hae*III were selected with a size-selection window of 264–464 bp. Sample processing followed the protocol of Sun et al. (2013), and final products were paired-end 150-bp sequenced using Illumina HiSeq (Illumina Inc.).

Reads were quality checked and those with low quality (*N* content > 10%, more than 50% of bases with quality values < 10) were removed using *fastp* (v 0.21.0, settings -q 10 -U 50 -y -g -Y 10 -e 20 -l 100 -b 150 -B 150; Chen et al. 2018). Reads were mapped to the *M. uniflorum* genome (Shirasawa et al. 2021, MUN_r1.1.fasta; available from https://horsegram.kazusa.or.jp/) using bwa (Li and Durbin 2009). Local realignment around indels was performed using RealignerTargetCreator and IndelRealigner in GATK (McKenna et al. 2010). SNPs were determined through two processes, using GATK UnifiedGenotyper with default settings (McKenna et al. 2010) and samtools mpileup (v1.9; (Li et al. 2009). SNPs called by both methods were considered reliable and retained using the SelectVariants package in GATK and outputted to a VCF (Variant Call Format) file.

We then removed sites with a Quality Score ≤ 30 or depth ≤ 20 using bcftools (https://samtools.github.io/bcftools/) and removed non-biallelic markers using VCFtools (ver. 0.1.16; Danecek et al. 2011). We then examined the effect of missing data on the number of SNPs retained, using the maximum number of missing samples 18 (40%), 9 (20%) and 5 (∼10%), again using VCFtools. For all three datasets we also removed SNPs in linkage disequilibrium using PLINK (settings 50 5 0.5; Purcell et al. 2007). For all three, bootstrapped Neighbour Joining (NJ) trees were produced by generating 1000 P-distance matrix using VCF2Dis (https://github.com/BGI-shenzhen/VCF2Dis), which were imported into FastME ver 2.0 (Lefort et al. 2015) to generate 1000 trees, then a consensus was computed with the phylip package *consense* (Felsenstein 2004). This was then visualized in iTOL (https://itol.embl.de/) and rooted with the outgroup samples.

A STRUCTURE analysis was performed to estimate the number of genetic clusters found in our samples (excluding the outgroup) on the dataset with a maximum of 9 missing samples. A STRUCTURE input file was generated using PLINK and used in STRUCTURE v2.3.4 (Pritchard et al. 2000). The analysis comprised a 20 000 iteration burn-in and then 50 000 MCMC repetitions and repeated 5 times per *K* (the putative numbers of clusters; from 1 to 8). To determine the optimal number of genetic clusters the Δ*K* method (Evanno et al. 2005) was performed using StructureSelector (https://lmme.ac.cn/StructureSelector/; Li and Liu 2018). Results were then plotted using the online server for CLUMPAK (Kopelman et al. 2015; http://clumpak.tau.ac.il/). An additional analysis for only the *M. uniflorum* samples (using the same settings to remove poor quality SNPs, those with >20% missing data and those in LD) was carried out based on the initial STRUCTURE analysis (see Results).

Nucleotide diversity, π (Nei and Li 1979), the fraction of sites with missing data and the inbreeding coefficient, F (Wright 1922), were calculated for each species using vcftools and then averaged across individuals within species. Two individuals were excluded based on the phylogenetic analyses (see Results).

## Results

### Morphological analysis

The phylogenetic analyses (see below) suggest that one accession of each species was apparently mislabelled and sample *M. axillare* 17142 appears to be a hybrid and so were excluded from the morphological analysis. Two further *M. uniflorum* accessions had insufficient plants for the morphological analysis. This left 14 *M. uniflorum* and 23 *M. axillare* samples for the stem colour and leaf size analysis. For the seed colour quantification and measurements, a further two accessions of each species had insufficient seed, leaving 12 *M. uniflorum* and 21 *M. axillare*.

**Figure 1:**
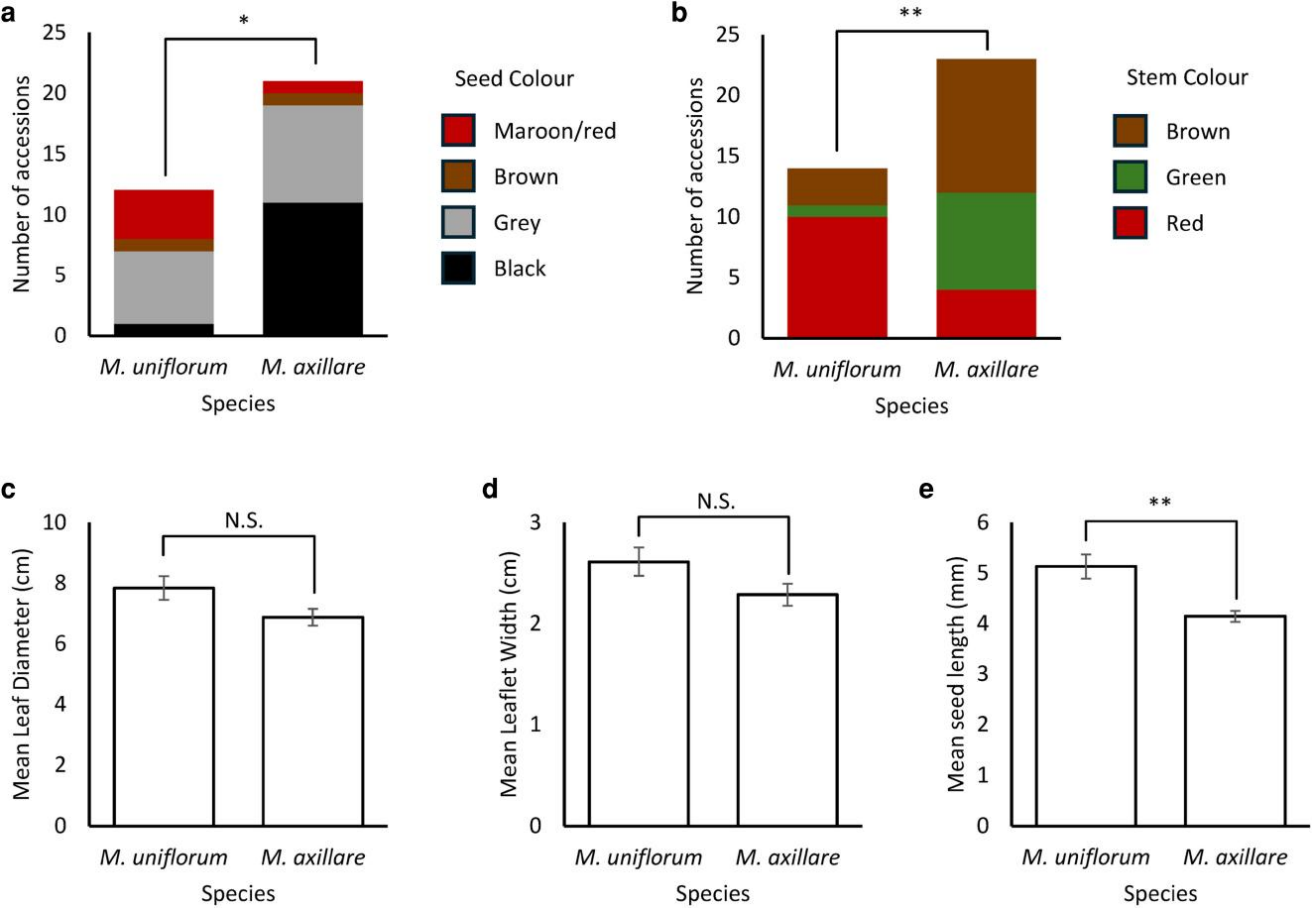
Comparison of morphological traits between the two species. (a) Dominant seed colour, (b) dominant stem colour, (c) leaf diameter, (d) leaflet width, (e) seed length. NS, not significant, *P < .05, **P < .01.

The distribution of seed colour was significantly different between the two species (Fisher's exact test *P*-value = .0199; Fig. 1a; Supplementary Figure S2). Red seeds were almost exclusively present and black almost completely absent in *M. uniflorum*. Grey and brown seeds were relatively common and relatively rare, respectively, but at similar frequencies in both species. The distribution of stem colour was also significantly different between the two species (Fisher's exact test *P*-value = .0044; Fig. 1b). Red stems are the most common in *M. uniflorum* and green and brown stems are most common in *M. axillare*.

Leaves and leaflets were generally larger in *M. uniflorum*, by about 12%, but this was not significantly different (*T*-tests: leaf diameter, *T* = 2.01, *df* = 25, *P* = .055, leaflet diameter, *T* = 1.84, *df* = 26, *P* = .077; Fig. 1c and d). Seed length was significantly different (*T*-test: *T* = 3.74, *df* = 15, *P* = .002) and was greater in *M. uniflorum* (5.132 ± 0.24 mm [SE]) than in *M. axillare* (4.143 ± 0.11 mm) (Fig. 1e). Because the *M. uniflorum* samples were split between wild (African) and cultivated (other locations) types (see Discussion), we compared seed size, and cultivated seeds were longer (5.474 ± 0.27 mm) than the wild seeds (4.447 ± 0.24 mm) (*T*-test: *T* = 2.83, *df* = 9, *P* = .020).

### Genetic analysis

An average of 4.8 M ± 0.23 M (SE) reads per sample were generated, of which over 93% mapped from each sample (Supplementary Table S1). Across samples the SLAF-seq analysis identified 317 439 SLAF tags, of which 213 336 were polymorphic and 2 992 601 SNPs were detected from these tags. Each sample generated an average of 104 K ± 3 K SLAF loci with an average depth of 18.8X ± 0.71 and 1.6 M ± 0.02 M SNPs (Supplementary Table S1).

After removing sites with a quality score (Q) < 30 and depth (DP) < 20, 1 475 140 SNPs were retained. We then filtered to only retain biallelic markers and to remove SNPs with missing data [either 18 samples missing (40%), 9 samples missing (20%), or 5 samples missing (∼10%)]. This resulted in retaining 268 992, 159 495, and 109 945 SNPs respectively. After removing SNPs in linkage disequilibrium, we were left with 53 284, 29 517, and 20 037 SNPs, respectively (Table 1).

**Table 1:** Number of SNPs for each dataset after applying different thresholds for quality control (quality, Q and depth, DP), the maximum number of samples missing a base call (MMIss), the number of alleles per SNP and whether SNPs in LD were removed (LD).

Q	DP	MMiss	*N* alleles	LD?	SNPs
No QC or trimming					2 992 601
30	20				1 475 140
30	20	18	2		268 992
30	20	9	2		159 495
30	20	5	2		109 945
30	20	18	2	LD trimmed	53 284
30	20	9	2	LD trimmed	29 517
30	20	5	2	LD trimmed	20 037

**Figure 2:**
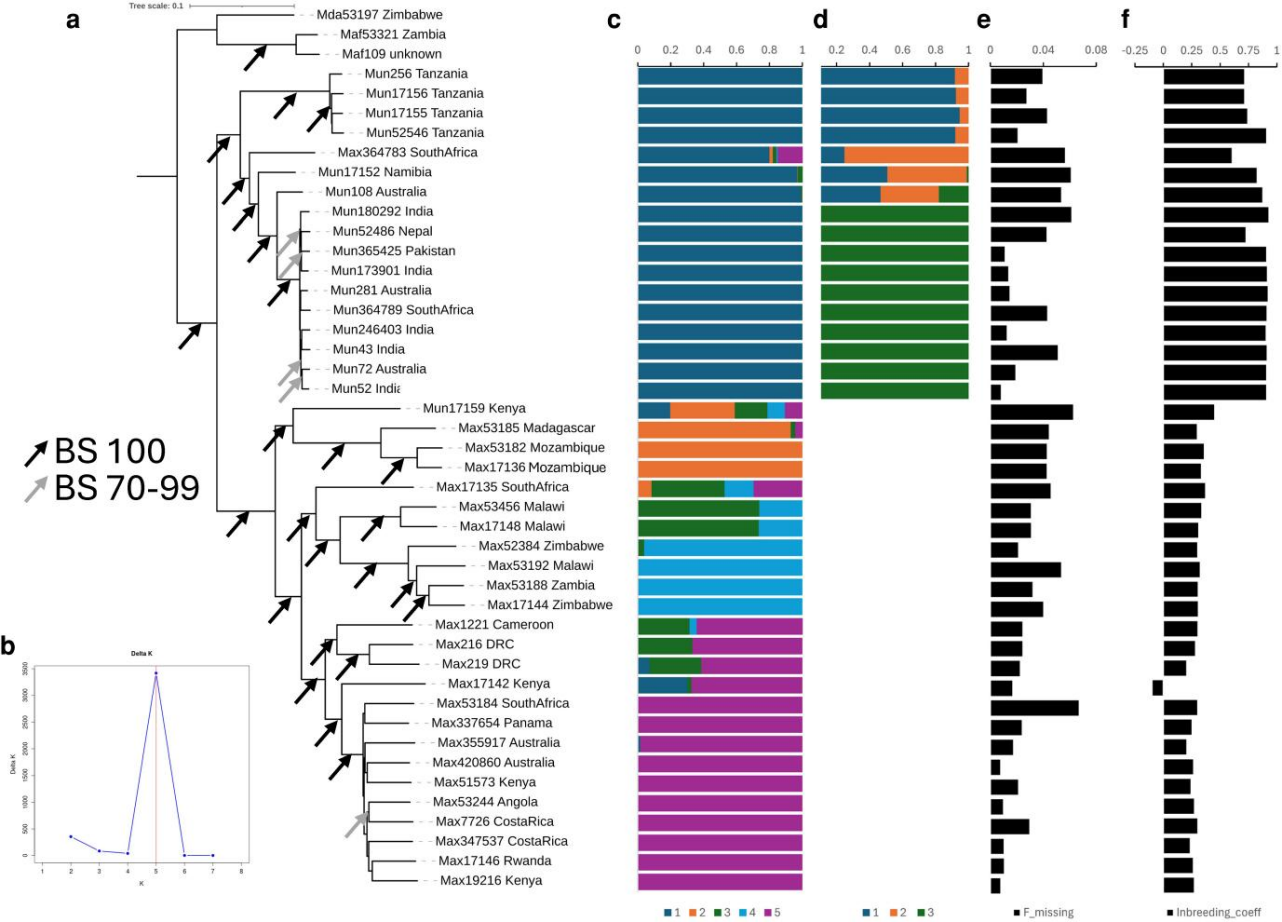
Genetic analysis of 42 samples of *Macrotyloma uniflorum* (mun), *M. axillare* (Max) and three outgroups (Maf and Mda). (a) NJ phylogenetic tree (bootstrap support is indicated by arrows, where no arrow means BS < 70%), (b) results of the ΔK analysis of the STRUCTURE results, (c) STRUCTURE analysis of sample clustering with *K* = 5 clusters, (d) STRUCTURE analysis of *M. uniflorum* with *K* = 3 clusters, (e) fraction of SNPs with missing data, (f) inbreeding coefficient, F.

NJ trees of these three datasets showed concordance apart from some minor differences in branches near the tips that were not bootstrap supported (Supplementary Figure S3). The dataset with the intermediate number of SNPs was therefore used going forward as a compromise between missing data and the number of SNPs for analysis (Fig. 2a). This dataset was used to examine genetic structure and clustering using STRUCTURE, which suggested that the most likely number of clusters (*K*) was five (Fig. 2b and c). Summarizing the phylogenetics and STRUCTURE analyses together, overall, there is clear distinction between the two species and evidence for subgroups within each, which are largely based on geography. For example, in *M. uniflorum*, one group of accessions is solely from Tanzania and the other contains accessions from Southern Africa and outside Africa. This suggests that the domesticated accessions (from India) are derived from this second gene pool.

Using *K* = 5, we find that 36 of the 42 ingroup samples have >70% membership to one cluster. Seventeen, 3, 2, 4, and 10 samples fall into clusters 1 to 5 (Fig. 2c). Cluster 1 (dark blue) comprises only *M. uniflorum* samples (including one presumed mislabelled sample, *M. axillare* 364 783) and clusters 2–5 comprise only *M. axillare* samples which were broadly separated by geography (Fig. 2c). Cluster 2 contains samples from Southeast Africa (Mozambique and Madagascar; orange), cluster 3 from Malawi (green), cluster 4 from South central Africa (Malawi, Zimbabwe, and Zambia; light blue), and cluster 5 (purple) from a broad distribution across Central Africa (Cameroon, Democratic Republic of the Congo, Kenya, Rwanda, and Angola) and countries outside Africa (Panama, Costa Rica, and Australia).

The six samples that were admixed under *K* = 5 included one *M. axillare* sample from Kenya at the base of cluster 5 which has partial membership to the *M. uniflorum* cluster, a potentially mislabelled sample which had partial membership to all clusters (*M. uniflorum* 17 159 close to *M. axillare* cluster 2) and a sample from South Africa (*M. axillare* 17 135) at the base of the group of samples in clusters 3 and 4. The other three samples that did not fit clearly into one cluster (*M. axillare* 1221, 216 and 219) are closely related to, but genetically divergent from, cluster 5, containing about 1/3 membership to cluster 3, again indicating samples group by geography (Fig. 2c).

The two subgroups in *M. uniflorum* resolved in the NJ trees are not resolved in the STRUCTURE analysis, therefore, an additional STRUCTURE analysis of only *M. uniflorum* was carried out which supported three clusters. This resolved the two groups from the NJ trees as distinct, with phylogenetically intermediate samples as potentially representing a third *M.* uniflorum cluster but with admixture (Fig. 2d).

We then compared genetic diversity in the two species. We excluded *M. axillare* 17 142 (unusual mixed ancestry) and *M. uniflorum* 17 159 (appears to be mislabelled and has high admixture). The other admixed individuals (and the mislabelled *M. axillare* 364 783) were included as they still clearly fit into one of the species groups. Consistent with the STRUCTURE analysis, nucleotide diversity (π) was greater in *M. axillare* [0.24968 ± 0.0009 (SE), *n* = 23] than *M. uniflorum* (0.09338 ± 0.0009, *n* = 17) (*T*-test: *T* = 123.01, *df* = 58 774, *P* = .000).

The estimate for π per species could be biased if missing data was greater in one species. Even though the average proportion of missing sites per individual was not significantly different between the two groups (*T*-test: *T* = 0.95, *df* = 30, *P* = .350), it was slightly greater in *M. uniflorum* (0.034) than *M. axillare* (0.029) (Fig. 2e). Therefore, we removed sites with any missing data in these 40 individuals (leaving 11 940 SNPs), but the pattern was upheld; π was over 2.5 times greater in *M. axillare* (0.25771 ± 0.0009 [SE]) than *M. uniflorum* (0.09435 ± 0.0009) (*T*-test: *T* = 83.07, *df* = 23839, *P* = .000).

Consistent with the reduced diversity, there were more monomorphic sites in *M. uniflorum* (6986/11 940; 58.5%) then in *M. axillare* (1334/11 940; 11.2%). In addition, the inbreeding coefficient (F) was significantly greater in *M. uniflorum* (0.846 ± 0.024 (SE)) than *M. axillare* (0.291 ± 0.043) (*t*-test: *T* = 21.25, *df* = 20, *P* = .000), indicating greater inbreeding and homozygosity in the former (Fig. 2f).

## Discussion

*Macrotyloma uniflorum* and *M. axillare* are minor/underutilised legumes with recognized potential as crops to supplement more widespread agriculture in the future changing climate, primarily because of heat and drought resistance and the nutritious nature of the seeds (Chahota et al. 2013, Aditya et al. 2019). Despite this, they remain underutilised. Genomes of the two species have been published (*M. uniflorum* (Mahesh et al. 2021, Shirasawa et al. 2021), *M. axillare* (Fisher et al. 2022)), with the latter being highly fragmented. Genome sequences should help to advance an understanding of the genetic basis of adaptive traits (Chapman et al. 2022), indeed attempts have been made to understand the genetic basis of yield and other traits in *M. uniflorum* (Sharma and Chahota 2024).

*Macrotyloma uniflorum* is cultivated in India, and southeast Asia for food and in Australia and Africa primarily as fodder (Chahota et al. 2013). It is ecologically adaptable, being cultivated at a range of altitudes and in areas of low to medium precipitation. *M. uniflorum* seeds are relatively high in protein (16%–30%) and contains several other micronutrients. Africa is the presumed centre of origin for *M. uniflorum*, with the Himalayas a secondary centre of diversity and likely the location of domestication (Arora and Chandel 1972, Fuller and Murphy 2018). It has additionally been suggested that *M. axillare* is the ancestor of *M. uniflorum* (Chahota et al. 2020, Mishra et al. 2025).

*Macrotyloma axillare* is less studied; it is grown as a fodder and is native to Africa but has been introduced to other parts of the world (Blumenthal and Staples 1993). It performs well as an intercrop as it can withstand droughts and shade as well as positively influencing the yield of the primary crop (Araújo et al. 2017, Yemataw et al. 2018). Perennial horsegram has a high yield per plant and has been reported as resistant to some diseases that *M. uniflorum* is susceptible to; therefore, it could serve as a donor of alleles for breeding a higher-yielding, disease-tolerant horsegram (Chahota et al. 2013).

There are still some important questions remaining about these two species which we aimed to address. First, to determine whether horsegram (*M. uniflorum*) and perennial horsegram (*M. axillare*) are reciprocally monophyletic, second, to analyse population structure and quantify genetic diversity within each species, and third, to compare morphological features to identify characters that can be used to differentiate the species.

The two are recognized as being closely related (Morris 2008, Mishra et al. 2025); however, a recent cpDNA phylogenetic analysis did not support the two species as reciprocally monophyletic (Fisher et al. 2022). Hybridization of the two species is difficult, and sterility is high in the F_1_s (Chahota et al. 2013, Mishra et al. 2025). Our genome-wide analysis of >29 000 SNPs demonstrates that the species are genetically distinct and reciprocally monophyletic. This conclusion, however, relies on two samples we included as being mislabelled. These two samples show some admixture in the STRUCTURE analysis, which could indicate hybridization and accession *M. uniflorum* 17 159 had small seeds and a brown stem, which are more typical of *M. axillare* (see results). We therefore presume that the species are reciprocally monophyletic; however, some hybridization may have occurred, giving rise to morphologically intermediate samples and/or mislabelling in the seed banks. Seedbank errors have been reported for older cultivars of lettuce (van de Wouw et al. 2011). We hypothesized that the two species could simply be annual and perennial forms of one species (as reported sometimes in rice). However, our data on genetic differentiation and previous data showing a strong barrier to hybridization between the species suggest this is not the case.

It has been reported that genetic diversity is being eroded in *M. uniflorum*, primarily because other crops are typically chosen and *M. uniflorum* is being abandoned (Chahota et al. 2013). Genetic studies support this, with genetic variation low in domesticated *M. uniflorum* (Mahesh et al. 2021, Shirasawa et al. 2021). No similar analysis of *M. axillare* has been carried out. It was recently shown that in the third cultivated *Macrotyloma* species (*M. geocarpum* (Harms) Maréchal & Baudet, Kersting’s groundnut) that genetic variation is significantly lower (about 90% lower) than other related legumes (Cheung et al. 2025).

Mahesh et al. (2021) and Sharma and Chahota (2024) found three genetic clusters within horsegram, and Shirasawa et al. (2021) found two. We found little evidence for population structure within South Asian accessions; however, substantial genetic variation when one considers African accessions too, especially a group of accessions from Tanzania that were well-supported as distinct. Verdcourt (1982) describes four varieties of *M. uniflorum* in Africa with two (var. *verrucosum* and var. *benadirianum*) only recorded from Tanzania and Kenya. The former an inland taxon and the latter is solely coastal, and both have smaller seeds that domesticated horsegram from India (Fuller and Murphy 2018). It seems our genetically distinct group of accessions from Tanzania likely corresponds to var. *verrucosum* given that three of the four samples are inland (with the fourth, the specific collection location is not recorded) and the seeds were smaller than the other African and South Asian material.

While it has been suggested that *M. axillare* is the ancestor of domesticated *M. uniflorum* (Chahota et al. 2020, Mishra et al. 2025), our analysis suggests that domesticated *M. uniflorum* (Indian accessions) is derived from within the wider *M. uniflorum* gene pool, potentially derived from accessions from Southern Africa (Namibia and South Africa) and not from East Africa (Tanzania). Cultivated horsegram has generally larger seeds and wider pods than the wild types found in Africa, a pattern which we found in our data. Wild *M. uniflorum* is recorded from India (Arora and Chandel 1972, Fuller and Murphy 2018), which places the domestication of horsegram in South Asia. Because our samples from India were all domesticated, we cannot confirm this with our data at present.

Within *M. axillare*, our analysis showed four genetic clusters which were largely coincident with geography, indicating that genetic drift and local adaptation *in situ* are the primary factors governing genetic diversity and that little movement of accessions has taken place. This is probably because *M. axillare* is still regarded as only useful as a forage or is even considered a weed; hence, there has been no incentive for seed movement or sharing. Verdcourt (1982) describes *M. axillare* var. *macranthum* primarily from Malawi and indeed one of our clusters (cluster 3), albeit only containing two samples, was distinct and from Malawi. The other accessions, in clusters 2, 4, and 5, all overlap with what Verdcourt described as var. *axillare*. Samples from outside Africa (Central America and Australia) are solely found in cluster 5 (purple in Fig. 2c).

Genetic variation was over 2.5 times greater in *M. axillare* than in *M. uniflorum* even after accounting for any biases in missing data. In addition, the *M. uniflorum* accessions showed a greater proportion of invariant loci and greater inbreeding coefficients than *M. axillare*, which is typical for inbred crops. It was previously reported that variation was low in domesticated *M. uniflorum* (see above). Indeed, in our analysis there is very little variation in the group of 10 primarily South Asian (Indian, Nepalese, Pakistani, one South African, and two Australian) accessions. Overall, it seems that the African accessions represent untapped generic diversity that could aid in the breeding of novel domesticated horsegram germplasm.

The species are similar in overall appearance and as their distributions overlap in Africa, from Angola, Zimbabwe, and Mozambique, to Tanzania and Kenya (Verdcourt 1982), being able to distinguish the species in the field would be useful. We show here that the distributions of seed and stem colour are distinct between the species, however, are not completely diagnostic. For example, red seeds are rare in *M. axillare,* and black seeds are rare in *M. uniflorum*, but not entirely absent. Other colours are common in both species. Seed colour is a trait that is often selected on during domestication or diversification because of colour preference (e.g. Coulibaly et al. 2020) or because of its relationship to taste or other characters (Abbo et al. 2014, Fernández-Marín et al. 2014). Singh et al. (2009) reported that light-coloured horsegram seeds germinate faster and the seedling have superior vigour than those from darker seed. Seed colour can also influence ability to partner with important soil bacteria, for example, related to nodulation in legumes (Jaiswal and Dakora 2024). The difference in seed colours between the species could therefore reflect direct or indirect human-induced selection for seed colour in domesticated horsegram.

Similarly, for stem colour, red stems are common in *M. uniflorum* and rare in *M. axillare*, with the reverse true for green and brown stems. Stem colour can be adaptive (Gould et al. 2010) but also exhibits plasticity in the field; therefore, the use of this character in field samples remains to be assessed. It should be reminded here that some accessions were heterogeneous for seed and stem colour; therefore, the dominant colour which we used for our analysis does not represent the only colour for some accessions.

Seed size differed between the species, and seeds were larger in *M. uniflorum.* However, seeds from the Tanzanian accessions of *M. uniflorum* are relatively small and similar to *M. axillare* samples; therefore, cannot be used as a diagnostic character. Together, a range of characters should be used in the field.

Overall, we find that the species are genetically distinct and at least partially morphologically distinguishable although more characters are needed to fully demonstrate this. We also recognize that relatively small sample sizes were examined and broader genetic and morphological analyses would allow further conclusions regarding the genetic variation and morphological distinctions within and between the species.

Genetic variation is higher in *M. axillare*, but contrary to previous work, there is substantial genetic variation in both species, which is largely structured by geography. The low variation in domesticated *M. uniflorum*, which has also been reported by others, might currently limit its adaptability, however, investigating the species from its native Africa, especially around Tanzania, could identify novel alleles for adaptation and other traits. These could be used in breeding programmes. *M. uniflorum* and *M. axillare* are drought and salinity tolerant, rich sources of protein and carbohydrates and grow well on poor quality soils (Prasad and Singh 2015). Furthermore, these species are valuable for medicinal uses in treating various conditions (Ravishankar and Priya 2012). Having uncovered population structure and novel genetic variation, especially in *M. uniflorum*, future studies identifying adaptive variation should consider incorporating these divergent samples in their analyses. We note here that the number of accessions available in seedbanks is only about 300 *M. uniflorum* and 150 *M. axillare*, with only 65% having geographic data attached. In addition, of the *M. uniflorum* samples, 70% are from India and Nepal, and not from Africa where the genetic variation is clearly greater. This suggests future collection prioritization in Africa would be worthy. Future research should also investigate the culture associated with these species, especially as they are known as ‘famine food’ or simply as weeds. This could be improved through advancing the distribution, production, marketing, and consumption of these two species (Wickens et al. 1989).

## Supplementary Material

plag003_Supplementary_Data

## Data Availability

Raw sequencing data has been deposited in NCBI SRA (Bioproject PRJNA1307957).
