# Supplementary material for: Genome-wide variation in and between two closely related underutilised horsegram species (Macrotyloma axillare and M. uniflorum, Fabaceae)
Source: AoB Plants. 2026 Jan 16;18(1):plag003. doi: 10.1093/aobpla/plag003 (PMC12893217; doi:10.1093/aobpla/plag003)
Supplement: plag003_Supplementary_Data [file plag003_supplementary_data.zip › SuppFigs.pdf]

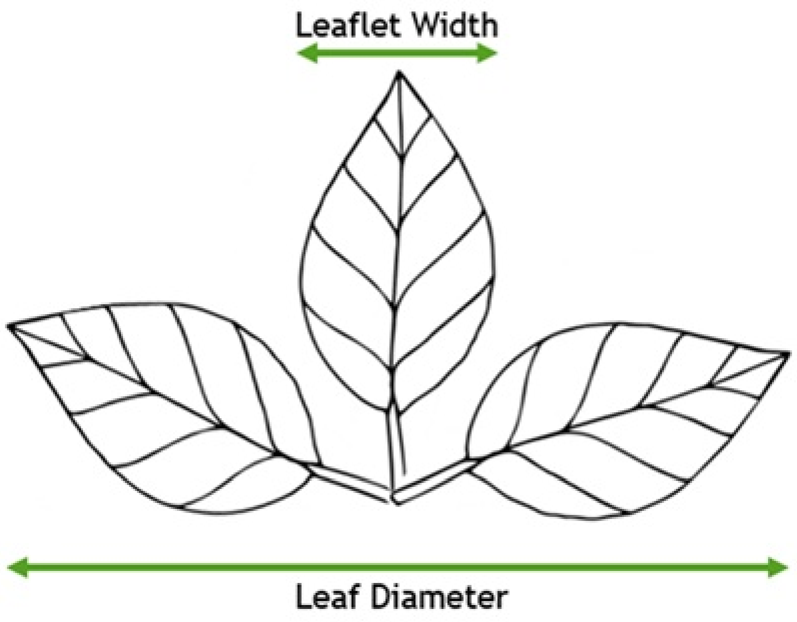

Supplementary Figure 1 – Diagram of leaf measurements made

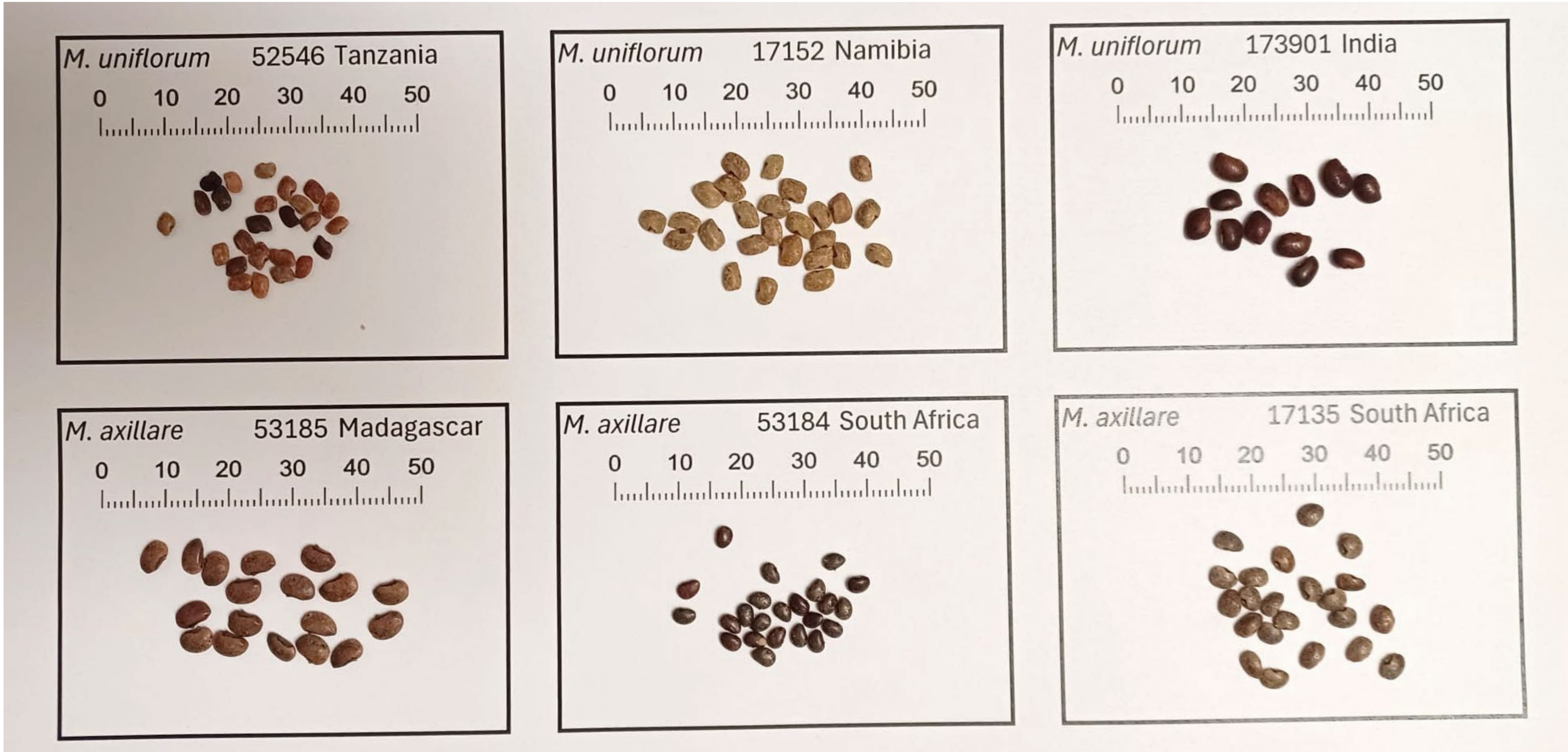

Supplementary Figure 2 – Examples of variation in seed size and colour

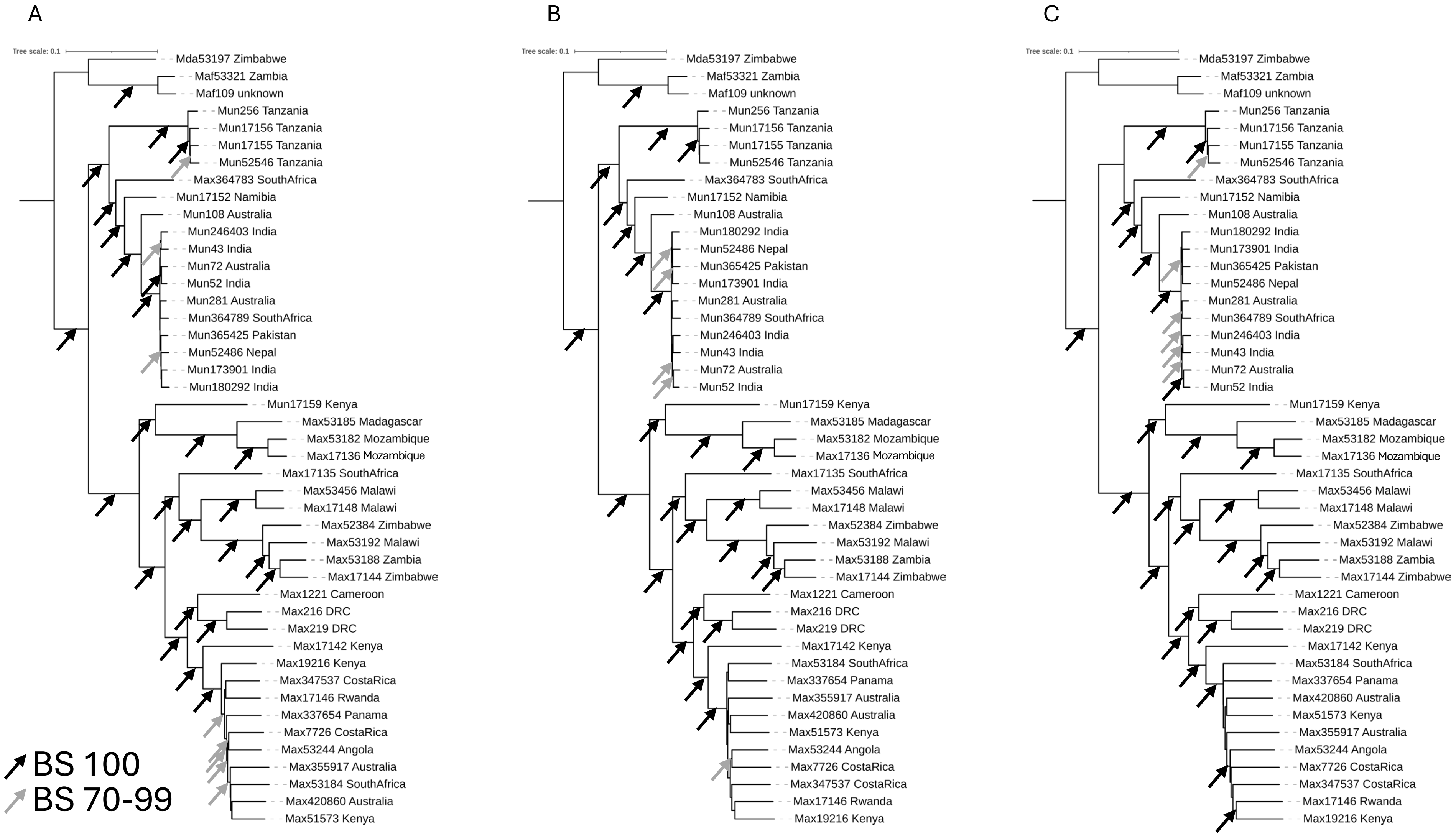

Supplementary Figure 3 – Neighbor Joining phylogenetic trees for (A) maximum 5 samples with missing data, (B) maximum 9 samples with missing data, and (C) maximum 18 samples with missing data. Bootstrap support is indicated by arrows, where no arrow means BS<70%.
